# Standards for Mobile Health–Related Apps: Systematic Review and Development of a Guide

**DOI:** 10.2196/13057

**Published:** 2020-03-03

**Authors:** Pere Llorens-Vernet, Jordi Miró

**Affiliations:** 1 Unit for the Study and Treatment of Pain-ALGOS Department of Psychology Universitat Rovira i Virgili Tarragona Spain

**Keywords:** mHealth, mobile apps, review, medical device, standards

## Abstract

**Background:**

In recent years, the considerable increase in the number of mobile health (mHealth) apps has made health care more accessible and affordable for all. However, the exponential growth in mHealth solutions has occurred with almost no control or regulation of any kind. Despite some recent initiatives, there is still no specific regulation procedure, accreditation system, or standards to help the development of the apps, mitigate risks, or guarantee quality.

**Objective:**

The main aim of this study was to propose a set of criteria for mHealth-related apps on the basis of what is available from published studies, guidelines, and standards in the various areas that are related to health app development.

**Methods:**

We used three sources of information to identify the most important criteria. First, we conducted a systematic review of all the studies published on pain-related apps. Second, we searched for health app recommendations on the websites of professional organizations. Third, we looked for standards governing the development of software for medical devices on the specialized websites of regulatory organizations. Then, we grouped and subsumed the criteria we had identified on the basis of their shared characteristics. Finally, the comprehensibility and perceived importance of the resulting criteria were evaluated for face validity with a group of 18 stakeholders.

**Results:**

We identified a total of 503 criteria from all sources, which, after close analysis, were grouped into eight different categories, including 36 important criteria for health apps. The resulting categories were *usability, privacy, security, appropriateness and suitability, transparency and content, safety, technical support and updates,* and *technology*. The results of the preliminary analysis showed that the criteria were mostly understood by the group of stakeholders. In addition, they perceived all of them as important.

**Conclusions:**

This set of criteria can help health care providers, developers, patients, and other stakeholders to guide the development of mHealth-related apps and, potentially, to measure the quality of an mHealth app.

## Introduction

### Background

Public health care systems worldwide are facing major challenges (eg, a shortage of resources and a steady increase in demand), which can make them increasingly unsustainable [1]. It is in this environment that what is known as mobile health (mHealth) is proving to be of key importance [2-4]. In the last few years, mHealth has undergone considerable development because of its potential to make health care more accessible and affordable for all [2,5-7].

However, mHealth solutions have grown exponentially with almost no control or regulation of any kind. In fact, very few of the health apps available have undergone a thorough validation process, and this causes a lack of confidence among health professionals [8,9]. For example, a recent review of the mobile apps available for chronic pain—which is one of the most prevalent health problems, with an enormous economic cost to individuals, families, and society [10]—highlighted that of the 283 apps available at the time, just a handful had undergone usability and validity tests [7]. This situation has been identified as preventing the field from improving and advancing [9].

In this so-called *strategic field*, progress depends not only on what each research group is doing but also on developing general standards and improving certification procedures [11,12]. There are some local and international initiatives to help in this process. For example, Catalonia approved a strategic action plan to support the development of mHealth (ie, *The Mobility Master Plan: mHealth solutions* [13]), which includes *AppSalut* [14], an accreditation system and guide [15] created to certify the quality of health– and social-related apps. At the international level, the European Commission published a Green Paper on mHealth [16] and launched a public consultation to identify potential barriers to and problems in the development of mHealth. Despite these initiatives, there is still no specific regulation procedure, accreditation system, or standards to help the development of apps, mitigate risks, and guarantee quality.

Therefore, the certification process is weighed down by the lack of clear standards to guide users through the different stages of the process. This is a problem not only for the safety of end users (ie, patients and health care professionals) but also for professional developers. Clearly, having a set of common criteria would be instrumental in helping the field to make progress in a consensual way and overcome potential risks for all stakeholders. There has been one recent attempt to develop a rating scale for mobile apps that could be used to help overcome this problem. Stoyanov et al [17] developed a scale (Mobile App Rating Scale [MARS]) to classify and rate the quality of mHealth apps. This scale was on the basis of a review of the papers published between 2000 and 2013, which contained explicit app-related quality rating criteria. However, this scale was created from a very narrow perspective for assessing already developed apps. That is to say, although the authors used information from studies on existing mobile apps, they failed to include information from other relevant sources that had been used, which would have increased the reliability and validity of their work (eg, standards governing the development of software for health or medical devices). Therefore, this scale does not seem to be suited for use by all stakeholders. Some more recent attempts to provide alternatives to assess mHealth apps also share some of these weaknesses (eg, developed for one specific group of stakeholders and using one specific source of information) [18,19].

### Objectives

The general aim of this study, then, is to go beyond what is already available and provide a standard for mHealth-related apps by studying the published studies, guidelines, and standards available in the field of health app development. In particular, we want to identify a set of criteria that are used, and which of these are strategic, so that they can be recommended and integrated into a general standard (ie, a guide) that can help the field move forward on solid grounds.

## Methods

### Procedure

We used three strategies to identify criteria. First, we conducted a systematic review of all the studies published on pain-related apps. Second, we searched the websites of professional organizations. Finally, we analyzed the standards governing the development of software for medical devices. Although these regulations are not specific for health apps, they can provide information of interest and complement the information collected.

### Information From the Systematic Review

For the systematic review, to address an otherwise unmanageable amount of information, we limited our search to mobile apps related to pain, one of the most prevalent health problems causing millions of visits to health care professionals. In so doing, we followed the Preferred Reporting Items for Systematic Reviews and Meta-Analysis guidelines [20] and searched the following databases: Web of Science, Scopus, PubMed, and ScienceDirect. We used the search terms (pain OR *ache) AND (smartphone* OR mobile) AND (app OR apps), and also hand searched the reference lists from relevant articles. Only peer-reviewed articles published in English or Spanish between 2008 (the release date of the first apps stores [21]) and December 2017 were included.

### Information on Websites From Professional Organizations

The second strategy consisted of searching websites from professional organizations that had guidelines and recommendations for health apps. We decided to focus our search on those regions where the mHealth market is most significant and, therefore, where these regulations are most likely to be found. According to a forecast of revenues of the world mHealth market [22], in 2017, the main mHealth markets by regions were Europe, North America, and Asia–Pacific, representing 30%, 28%, and 30% of the world market, respectively. In addition, the 15 most attractive countries for digital health solutions in 2017 were located in the regions mentioned above [23]. Therefore, we limited our search to those three areas. Again, and to make the search feasible, we limited our analysis to those countries that are the main markets in each region. In Europe, we included the United Kingdom and Spain as they had the same importance in terms of mHealth markets; in North America, the United States; and in Asia–Pacific, Australia.

### Information From Standards Governing the Development of Software for Medical Devices

Finally, in our search for information, we also searched for standards governing the development of software for medical devices on the specialized websites of regulatory organizations. To conduct this search, we also focused on the regions and countries where the mHealth market has been shown to be most significant, as described above. In this analysis, we focused the search on those standards with criteria related to health apps, added to our list only those criteria that are specific to health apps, and left all others out of our scrutiny (eg, protection against radiation and chemical properties).

### Development of a Common Set of Criteria and Categories

We first compiled a list of the criteria identified in (1) published studies, (2) guidelines, and (3) standards governing the development of software for medical devices. Next, we grouped the criteria in categories on the basis of their shared characteristics. That is, each criterion was closely analyzed to identify what its general purpose was (eg, the criterion *the functionality is adapted to the purpose of the app* was considered related to usability, and this opened a group or category that was labeled *usability*). All criteria underwent the same scrutiny. In the case that no category existed, a new one was created and labeled. If the category already existed, then the criterion was subsumed under that existing category. As a result of this analysis, we obtained a list of unique criteria classified into categories according to their similarity.

### Preliminary Analysis of the Set of Criteria

The resulting set of criteria underwent a preliminary analysis of their face validity by asking stakeholders to report on the comprehensibility and perceived importance of all the criteria. Specifically, in this analysis, we requested the collaboration of a group of individuals from different groups of stakeholders (ie, final users, potential patients, health care professionals, and developers or engineers). Final users or potential patients and health care professionals were approached by the authors while at the university hospital (while they were visiting for a health checkup and while at work, respectively). Engineers were professors or technicians working at the university. Before the participation of stakeholders, we first requested and obtained permission from the Ethics Committee of the School of Education Sciences and Psychology for the study procedures. Participants had to sign a consent form. All were asked to respond to two questions in relation to each criterion: (1) “Do you understand the criterion?” and (2) “How important is this criterion for a health-related mobile application?” The first one was responded with *yes*, *no*, or *partially* to the question, whereas the second one was to be responded by providing a number that best represented the importance of the criterion, between 0 (*not important at all*) to 10 (*utmost important*).

## Results

### Information From the Systematic Review

**Figure 1:**
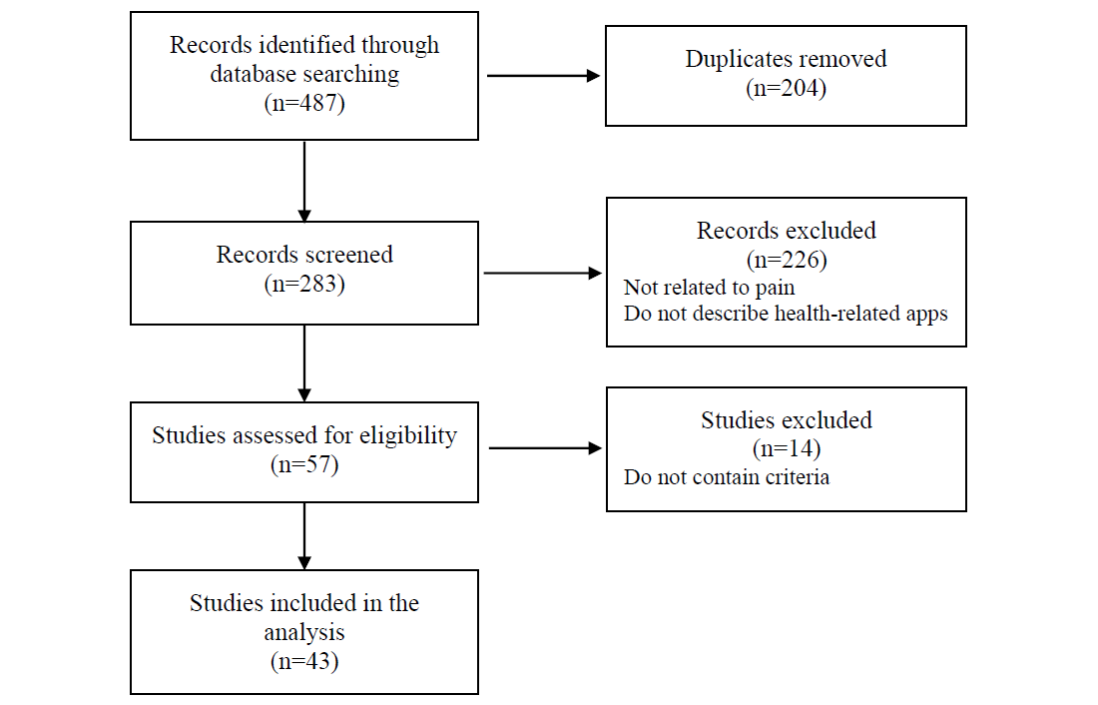
Flowchart of systematic review selection process.

Our review of the scientific databases identified 283 nonduplicated papers. Of these, only 43 were of interest for our purposes. Studies that were not related to pain or that did not describe health-related apps were deemed irrelevant, and not included in the analysis (see Figure 1). In this search, 168 criteria were identified (the full list is provided in Multimedia Appendix 1).

### Information From Websites of Professional Organizations

Following the planned strategy, we found just 4 organizations that had developed guidelines and recommendations for health-related apps. Of these, 3 were of national coverage—Andalusian Agency for Healthcare Quality (Spain), TIC Salut Social Foundation (Spain), and National Health Service (United Kingdom)—and 1 of supranational or international coverage, the European Commission (European Union [EU]). No similar information was found in the other searched regions. From each of the guidelines, we collected only the criteria that were specifically related to mobile phone apps and discarded the criteria related to other technologies (eg, wearables and websites): Andalusian Agency for Healthcare Quality, 31 criteria; European Commission, 58 criteria; National Health Service, 78 criteria; and TIC Salut Social Foundation, 115 criteria (see Multimedia Appendix 1).

### Information From Standards Governing the Development of Software For Medical Devices

As planned, in our search of the main mHealth markets, we also looked at the specialized websites of regulatory organizations and searched through standards in the regulations of medical devices. In so doing, we found just two standards that were of interest: (1) *Mobile Medical Applications: Guidance for Food and Drug* (the United States) and (2) *Regulation of medical software and* *mobile medical “apps”* (Australia). In this analysis, we added to our list only those criteria that were specific to health apps and left all others out of our scrutiny (eg, protection against radiation and chemical properties). We identified 42 and 11 criteria, respectively (see Multimedia Appendix 1).

### Development of a Common Set of Criteria and Categories

Then, the set of criteria were grouped in categories according to their similarity. That is to say, the criteria of the same class were grouped and subsumed together (see Table 1), resulting in eight categories. The categories were the following: *usability* (this includes criteria that are related to user experience), which contained eight criteria; *privacy* (ie, criteria related to data protection, compliance with the law, and treatment of users’ data), which contained six criteria; *security* (ie, criteria related to cybersecurity, encryption mechanisms for the storage and transmission of data, and measures against vulnerabilities), which contained four criteria; *appropriateness and suitability* (ie, criteria related to the adaptation of the app for the benefit of the targeted user), which contained three criteria; *transparency and content* (ie, criteria related to the sharing of information in relation to the development of the app), which contained five criteria; *safety* (ie, criteria related to the identification and prevention of harm to end users), which contained two criteria; *technical support and updates* (ie, criteria related to helping the user to solve problems in using the app), which contained four criteria; and *technology* (ie, criteria related to the proper functioning of the app), which contained four criteria (see Table 1).

### Preliminary Analysis of the Set of Criteria

A total of 18 individuals participated: 7 final users or potential patients, 6 health care professionals, and 5 developers, all of whom were approached and consented. Participants’ age ranged from 18 to 53 years, with an equal distribution of females and males in the sample. At the time of participation, all were attending school or working. They all had experience with mobile phones and in using mobile apps.

The results of this analysis are summarized in Table 1, which includes information about the percentage of participants within each group that understood the criteria, and the mean of the perceived importance of each one.

The criteria were understood by most of the participants in the three groups. However, at least one participant in one or more groups reported being unsure about the exact meaning. All the issues were related to the use of technical vocabulary or lack of some very specific (technical) knowledge; nevertheless, with additional explanation, the issues were solved. In addition, all criteria were perceived as important; 7 (on a 0-10 numerical rating scale) was the lowest rating received by any criterion, and most ratings were between 8 and 10 (Table 1 summarizes the information).

**Table 1:** Comprehensibility and perceived importance of the criterion by stakeholders.

Category and criterion		Comprehension, n (%)			Perceived importance (0-10)		
		Patients (N=7)	Clinicians (N=6)	Engineers (N=5)	Patients	Clinicians	Engineers
**Usability**							
	The app has been tested by potential users before being made available to the public.	7 (100)	5 (83)	5 (100)	8.9	8.6	9.4
	It has instructions or some kind of assistance for use.	6 (86)	6 (100)	5 (100)	10	8.5	7
	It is easy to use (ie, navigation is intuitive).	7 (100)	6 (100)	5 (100)	9.6	9.3	9
	It follows the recommendations, patterns, and directives in the official manuals of the different operating systems (Android, iOS, or others).	7 (100)	5.5 (92)	4 (80)	7.5	7	7.9
	The interface design follows the same pattern. That is, all graphic elements (typographies, icons, and buttons) have a consistent appearance. The function of each element (navigation menu, lists, and photo gallery) is clearly identified.	7 (100)	6 (100)	5 (100)	9	8.3	8.8
	The functionality is adapted to the purpose of the app.	7 (100)	5.5 (92)	5 (100)	9.6	8.5	8.8
	The information of the app must be able to be accessed in the shortest possible time. All users must be able to access all resources regardless of their capabilities.	7 (100)	5.5 (92)	5 (100)	8.3	8.6	8.4
	The app can be consulted in more than one language. All languages adapt appropriately to the content interface.	7 (100)	5.5 (92)	5 (100)	8.3	7.4	7.2
**Privacy**							
	The app gives information about the terms and conditions of purchases in the app and personal data recorded.	7 (100)	6 (100)	5 (100)	9.5	8.5	9
	It gives information about the kind of user data to be collected and the reason (the app must only ask for user data that is essential for the app to operate). It gives information about access policies and data treatment and ensures the right of access to recorded information. It describes the maintenance policy and the data erasure procedure. It gives information about possible commercial agreements with third parties.	7 (100)	5.5 (92)	5 (100)	9.6	8.8	9.4
	It guarantees the privacy of the information recorded. It requires users to give their express consent. It warns of the risks of using the app.	7 (100)	6 (100)	5 (100)	9.9	9.2	9
	It tells users when it accesses other resources of the device, such as their accounts or their social network profile.	7 (100)	6 (100)	5 (100)	9.3	8.3	9.4
	It takes measures to protect minors in accordance with the current legislation.	7 (100)	5.5 (92)	5 (100)	8.7	9.7	8.2
	Confidential user data are protected and anonymized, and there is a privacy mechanism so that users can control their data.	7 (100)	6 (100)	5 (100)	9.6	9.2	9.4
**Security**							
	The app has encryption mechanisms for storing, collecting, and exchanging information. It has password management mechanisms.	7 (100)	6 (100)	5 (100)	9.9	8.3	8.6
	The cloud services used have the relevant security measures. It states the terms and conditions of cloud services.	7 (100)	6 (100)	5 (100)	9.5	8.2	8.4
	The authorization and authentication mechanisms protect the users’ credentials and gives access to their data. It limits access to data that is only necessary for the user.	7 (100)	6 (100)	5 (100)	9.6	7.5	9.6
	It detects and identifies cybersecurity vulnerabilities, possible threats, and the risk of being exploited. It applies the appropriate security measures to cybersecurity vulnerabilities in the face of possible threats.	7 (100)	5.5 (92)	5 (100)	9.3	8.3	8.4
**Appropriateness and suitability**							
	The end users for whom the app is designed are explicitly indicated or actually intuitable (the name identifies the app) to the audience to whom it is set out.	7 (100)	6 (100)	5 (100)	8.2	7.7	8.4
	The benefits and advantages of using the app are explained.	7 (100)	6 (100)	5 (100)	9	7	7.5
	The app has been validated or created by experts (eg, a group of specialized professionals, a health organization, or a scientific society).	6.5 (93)	5.5 (92)	5 (100)	8	9.7	9
**Transparency and content**							
	The app identifies the authors of the content and their professional qualifications.	7 (100)	6 (100)	5 (100)	8	8.5	8.4
	It gives transparent information about the owners’ identity and location.	7 (100)	6 (100)	5 (100)	7.2	8.2	8.4
	It gives information about its sources of funding, promotion and sponsorship, and possible conflicts of interests. Any third parties or organizations who have contributed to the app development are clearly identified.	7 (100)	6 (100)	5 (100)	7.5	7	7
	It uses scientific evidence to guarantee the quality of the content. It is based on ethical principles and values.	7 (100)	6 (100)	5 (100)	10	9.3	9
	The sources of the information are indicated. Concise information is given about the procedure used to select the content.	7 (100)	6 (100)	5 (100)	8.2	7	8
**Safety**							
	The possible risks to users are identified. Users are warned that the app does not intend to replace the services provided by a professional.	7 (100)	6 (100)	5 (100)	9.5	8.9	8.6
	Potential risks for users caused by bad usage or possible adverse effects are explained.	7 (100)	6 (100)	5 (100)	8.5	8.5	8.6
**Technical support and updates**							
	It gives a warning if updates modify or affect how the app functions. It gives a warning if updates can influence insensitive data.	7 (100)	6 (100)	5 (100)	8.5	7.2	7
	Frequent security updates are guaranteed. Every time an update of a third-party component is published, the change is inspected, and the risk evaluated.	7 (100)	5.5 (92)	4.5 (90)	8.2	8.2	7
	The frequency with which the content of the app is revised or updated is shown.	7 (100)	6 (100)	4.5 (90)	7.7	7	7
	Users have support mechanisms (email, phone, and contact form) for solving doubts, problems, or issues related to the health content, and technical support.	7 (100)	6 (100)	5 (100)	9.2	9	8.4
**Technology**							
	It works correctly. It does not fail during use (eg, blocks). Functions are correctly retrieved after context changes (eg, switch to another app and return), external interruptions (eg, incoming calls or messages), and switching off the terminal.	7 (100)	6 (100)	5 (100)	9.5	8.5	9
	It does not waste resources excessively: battery, central processing unit, memory, data, or network.	7 (100)	6 (100)	5 (100)	8.9	7.8	8.2
	It can work in flight mode and deal with network delays and any loss of connection.	7 (100)	5.5 (92)	5 (100)	7.9	7.2	7
	It supports multiple versions of data structures or formats (eg, to support different operating systems).	6.5 (93)	5.5 (92)	4.5 (90)	8	7	7

## Discussion

### Principal Findings

To the best of our knowledge, this study is the first one to provide a guide to help with the design, development, and analysis of mHealth-related apps, in the form of a list of criteria and categories. This guide is based on an in-depth analysis of criteria that have been described in published studies on pain-related mHealth apps, guidelines, and best practices, as designated on the websites of professional and regulatory organizations from the most significant regions and countries of the world mHealth market.

In this study, we identified 36 criteria that are important to the design, development, and analysis of mHealth-related apps, which were grouped and subsumed into eight categories according to their similarity: (1) *usability* (ie, the app must be adapted to the targeted population), (2) *privacy* (ie, compliance with the law and treatment of users’ data), (3) *security* (ie, data protection, authorization mechanisms, and detection of vulnerability), (4) *appropriateness and suitability* (ie, the benefits and advantages for the end users are explained), (5) *transparency and content* (ie, scientific evidence and sources information), (6) *safety* (ie, the potentiality of risk to end users), (7) *technical support and updates* (ie, there is a policy about the maintenance of the app after it has been launched), and (8) *technology* (ie, the app works smoothly and does not fail abruptly).

In addition, this set of criteria underwent a test, and the preliminary data have shown that the criteria are understood by potential users. Furthermore, they have been reported to be of high importance by the group of stakeholders. Of particular importance (ie, a criterion that was valued as 9 or higher by all stakeholders groups on a 0-10 numerical rating scale) were the following: (1) *It is easy to use* (ie, navigation is intuitive); (2) *It guarantees the privacy of the information recorded. It requires users to give their express consent. It warns of the risks of using the app*; (3) *Confidential user data is protected and anonymized, and there is a privacy mechanism so that users can control their data*; and (4) *It uses scientific evidence to guarantee the quality of the contents. It is based on ethical principles and values*.

Our work improves previous proposals as it brings together information from a variety of internationally relevant sources (ie, research studies, data from websites of professional organizations, and standards governing the development of software for health or medical devices), whereas available ones have been developed narrowly, mostly using just one source (eg, studies on mobile apps [17]), sometimes using data of unknown scientific value (ie, mobile apps available on Web-based stores that have not undergone usability or validity studies [19]). This might be responsible, at least in part, for missing information in available guides. For example, in the case of the MARS [17], which is one of the most used rating systems, authors have failed to include some very basic items on their scale. Of particular concern are the issues of privacy and security of users’ information, which are not on the scale. The protection of users’ information is mandatory by law, so it is fundamental for all scales to include this as part of an integral evaluation of a mobile app. Likewise, the scale attaches little importance to whether an app is evidence-based or trialed in well-controlled studies. For example, a recent study that used MARS [24] to assess the quality of pain-related mobile apps showed that of the 18 apps, the 2 that had been scientifically tested were given the worst scores on the scale, and 1 of these had already been awarded a seal of quality from a public agency. It does seem that with MARS, the so-called commercial apps are better rated than those that have been scientifically tested and shown to provide valid and reliable information. This goes against the current trend in the area, which is seeking apps that have been scientifically tested and designed on the basis of evidence [25-27]. Furthermore, Salazar et al [24] showed that when MARS is used, an app developed with a highly specific objective in mind (eg, to measure pain intensity) will show lower scores (and will, therefore, be assumed to provide worse measurements) simply because of its specificity. Finally, the questions on the rating scale developed by Stoyanov et al [17] were mostly written to be answered by end users and require responses that are highly subjective or cannot be answered by a person who is not an expert in the field (eg, “Is app content correct, well written, and relevant to the goal or topic of the app?”).

In addition, the preliminary data on the comprehension of the criteria showed that they can be understood by different profiles of stakeholders, as intended. However, a few of them reported having problems with some criteria, which were solved after giving additional explanations. Therefore, it is important that the information is presented with the least technical wording possible to facilitate comprehension. Nevertheless, additional studies with more participants to validate and extend the findings are warranted.

The resulting guide with this set of criteria describes the standard to follow, identifies the main categories of criteria, and provides stakeholders with a systematic approach by which they can determine the general requirements of a mobile app if it is to be considered of high quality. An app that meets these criteria is one that will provide users with the greatest security and confidence in performance and the objectives being fulfilled.

### Limitations

This study has limitations that should be considered when interpreting the results. First, our search strategy was limited to papers written in English or Spanish, pain-related apps, and guidelines and standards published in specific regions and countries. We made these choices because it was what we could feasibly do, but we cannot be certain that we have included *all* the important criteria. For example, some issues could be seen as more important by developers of pain-related apps compared with developers of apps related to sexual health (eg, pain-related apps are biased toward treatment rather than diagnosis; pain-related apps may primarily be targeted at the patient, rather than health professionals or carers). We analyzed the studies on pain-related apps, and the information was combined with that from the most important markets for mHealth apps and on guidelines and standards available, as a way to complement each other and solve the potential limitations. Nevertheless, the final result of our analysis is limited in ways that we cannot completely foresee. Therefore, future studies on the validity and reliability of this set of criteria are warranted. Second, the comprehension test was conducted with a small group of 18 individuals from three groups of stakeholders. Although the number of participants was enough for a preliminary analysis, the sample is not representative. Thus, additional studies, including samples with more participants, are needed. Despite these limitations, this study provides important new information to help advance the field.

### Conclusions

This set of criteria can be readily used by health care providers, engineers and developers, researchers, patients, and regulators. The data have shown them to be comprehensible and of importance for a group of stakeholders. Nevertheless, future studies will have to empirically test the validity, reliability, and suitability of this set of criteria. Furthermore, they should be analyzed in terms of their significance to all stakeholders so that the set of criteria could also be used as a guide to the quality of the apps by all interested parties.

## Appendix

Multimedia Appendix 1 Full list of criteria.

## Abbreviations

EU: European Union
MARS: Mobile App Rating Scale
mHealth: mobile health
