# Supplementary material for: Standards for Mobile Health–Related Apps: Systematic Review and Development of a Guide
Source: JMIR Mhealth Uhealth. 2020 Mar 3;8(3):e13057. doi: 10.2196/13057 (PMC7078629; doi:10.2196/13057)
Supplement: Multimedia Appendix 1 [file mhealth_v8i3e13057_app1.doc]

**Table S1. List of all criteria**

**Published studies**

| 1 | All users agreed to the collection of data presented in this publication by signing the terms and conditions for use |
| --- | --- |
| 2 | Protection of personal data |
| 3 | Scientific framework |
| 4 | Scientific evaluation |
| 5 | Anonymized data |
| 6 | Evidence-based content, theoretical grounding |
| 7 | Health care professional involvement |
| 8 | Involved end users |
| 9 | Health care professionals were involved |
| 10 | Evidence-based content |
| 11 | All data were saved on a secure encrypted password-protected server |
| 12 | Limited access to data |
| 13 | App has measures in cybersecurity |
| 14 | Feasibility app |
| 15 | Safe application for the user |
| 16 | The application is effective with its purpose |
| 17 | Easy to use |
| 18 | Reliable and secure electronic data collection |
| 19 | Usability testing |
| 20 | User-friendliness |
| 21 | Clarity of information provided |
| 22 | Usability testing |
| 23 | Effectiveness |
| 24 | Provide sources information |
| 25 | App reliability |
| 26 | App validation |
| 27 | Effectiveness |
| 28 | Usability testing |
| 29 | App acceptability |
| 30 | Validity of application |
| 31 | Reliable data collection |
| 32 | Usability testing |
| 33 | Intuitive design |
| 34 | Acceptability of application |
| 35 | The functionalities of the application are efficient |
| 36 | Scientific content |
| 37 | Useful utilities |
| 38 | Accessibility |
| 39 | Evidence-based content |
| 40 | The functionalities are adapted to the user |
| 41 | App acceptability |
| 42 | Usability testing |
| 43 | Implement data protection |
| 44 | Informed Consent |
| 45 | Data confidentiality |
| 46 | Reliable data collection |
| 47 | Scientific evidence |
| 48 | Intuitive design |
| 49 | Usability Testing |
| 50 | The application must be tested |
| 51 | Effectiveness |
| 52 | Validated by healthcare professionals |
| 53 | Native design |
| 54 | App more intuitive |
| 55 | User-centred design |
| 56 | Information evidence-based |
| 57 | Usability testing |
| 58 | Content will be evaluated by experts |
| 59 | Ongoing technical support |
| 60 | Evidence-based interventions |
| 61 | The functionalities are adapted according to the type of app |
| 62 | Well-targeted to audience |
| 63 | Functionality is adapted |
| 64 | Easy to learn |
| 65 | Intuitive graphic design |
| 66 | Quality and quantity of information |
| 67 | Offer information about the credibility of developer |
| 68 | Scientific evidence base |
| 69 | Ease of use |
| 70 | Evidence-based practices |
| 71 | Health care professional involvement |
| 72 | Usability testing |
| 73 | Easy to use |
| 74 | Error-free |
| 75 | Effectiveness |
| 76 | Efficiency |
| 77 | The application is adapted to the purpose |
| 78 | Identify possible risks |
| 79 | Standardized instructions |
| 80 | Secure data transmission |
| 81 | Intuitive design |
| 82 | Usability testing |
| 83 | App acceptability |
| 84 | App reliability |
| 85 | Evidence-based content |
| 86 | The application is adapted to the purpose |
| 87 | Usability testing |
| 88 | The app is effective for its purpose |
| 89 | Usability testing |
| 90 | Provide education, support and documentation |
| 91 | Effectiveness |
| 92 | Evidence-based treatments |
| 93 | Health care professionals are involved |
| 94 | App effectiveness |
| 95 | Feasibility app |
| 96 | Usability testing |
| 97 | Easy to use |
| 98 | Feasibility app |
| 99 | Few technical difficulties |
| 100 | Incorporate functionalities that facilitate achieving the purpose of the app, such as gamification |
| 101 | Intuitive design |
| 102 | The application must be accessible to any user |
| 103 | Informed Consent |
| 104 | Effective functionalities for its purpose |
| 105 | Standardized instructions |
| 106 | Healthcare professionals involvement |
| 107 | Intuitive interface design |
| 108 | Usability testing |
| 109 | Effectiveness |
| 110 | App Feasibility |
| 111 | Expert recommendations |
| 112 | Usability testing |
| 113 | Usability testing |
| 114 | Feasability |
| 115 | Native graphic design |
| 116 | Robust offline functionality |
| 117 | Informed consent |
| 118 | Implement data protection |
| 119 | The application is effective with its purpose |
| 120 | Informed consent |
| 121 | App Reliability |
| 122 | Usability testing |
| 123 | App feasibility |
| 124 | Easy to use |
| 125 | Health care professionals are involved |
| 126 | Used without an Internet connection |
| 127 | The functionalities is adapted to the user |
| 128 | Well-targeted to audience |
| 129 | Error-free |
| 130 | Standardized instructions |
| 131 | Health care professionals involvement |
| 132 | The application is accessible to any user |
| 133 | Health care professionals are involved |
| 134 | Privacy conditions are indicated |
| 135 | Accessibility |
| 136 | The application must be validated |
| 137 | Implement security measures for data |
| 138 | Scientific rigor |
| 139 | Indicate the authors of the contents |
| 140 | Intuitive iInterface design |
| 141 | Usability testing |
| 142 | Clinician involvement |
| 143 | Researchers involvement |
| 144 | Easy to use |
| 145 | Adapted functionalities |
| 146 | Intuitive design |
| 147 | Protect personal data |
| 148 | Well-validated |
| 149 | Incorporate functionalities that facilitate achieving the purpose of the app, such as gamification |
| 150 | Intuitive design |
| 151 | Usability testing |
| 152 | Ease of app use |
| 153 | Informed consent |
| 154 | Well-targeted to audience |
| 155 | The information given related to health must be validated |
| 156 | Health care professionals are involved |
| 157 | Health care professional involvement |
| 158 | Incorporate privacy consent |
| 159 | Intuitive design |
| 160 | Health care professionals involvement |
| 161 | Intuitive design |
| 162 | Health care professionals are involved |
| 163 | Usability testing |
| 164 | App reliability |
| 165 | Easy to use |
| 166 | The built-in functionalities are useful for the user |
| 167 | Scientific content |
| 168 | The application is effective with its purpose |

**Guidelines**

Andalusian Agency for Healthcare Quality (Spain)

| 1 | The health App clearly defines its functional reach and its purpose, identifying the target groups of information and the aims pursued regarding these groups. |
| --- | --- |
| 2 | The health App follows the Principles of Universal Design, as well as reference accessibility standards and recommendations. |
| 3 | The health App follows the recommendations, patterns and directives included in the official manuals of the different platforms. |
| 4 | The health App has been tested by potential users before its availability to the public. |
| 5 | The health App adapts itself to its target audience. |
| 6 | The health App offers transparent information about its owners’ identity and location. |
| 7 | The health App offers information about its funding sources, promotion and sponsorship, as well as about possible conflicts of interests. |
| 8 | The health App identifies the authors of its content and their professional qualification. |
| 9 | The health App includes the date of the last revision made in the published material. |
| 10 | The health App warns of those updates which modify or influence the functioning of health-related content, as well as other sensitive data. |
| 11 | The health App is based on one or more reliable information sources and takes into account the available scientific evidence. |
| 12 | The health App offers concise information about the procedure used in order to select its content. |
| 13 | Health App is based on ethical principles and values. |
| 14 | The possible risks for patient safety caused by the use of the health App are identified. |
| 15 | The known risks and adverse events (near misses) are analysed, and the convenient actions start to be developed. |
| 16 | The health App has a support system about its use. |
| 17 | The health App offers a contact mechanism for technical support with an assured and fixed response time. |
| 18 | The health App informs about the terms and conditions on its products and services’ commercialisation. |
| 19 | The health App makes an efficient use of communications bandwidth. |
| 20 | The health App warns of the use of advertisement mechanisms and allows deactivating or skipping it. |
| 21 | Before downloading and installing, the health App informs about the kind of user’s data to be collected and the reason, about the access policies and data treatment, and about possible commercial agreements with third parties. |
| 22 | The health App clearly describes the terms and conditions about recorded personal data. |
| 23 | The functioning of the health App preserves privacy in the recorded information, collects express consents granted by users, and warns of risks coming from the use of online mobile health Apps. |
| 24 | The health App ensures pertinent security measures when users’ health information or sensitive data has to be collected or exchanged. |
| 25 | The health App informs the users when it has access to other resources of the device, to users’ accounts and to profiles in social networks. |
| 26 | The health App ensures the right of access to recorded information and the updates regarding changes in its privacy policy. |
| 27 | The health App has measures regarding minors’ protection in accordance with the current legislation. |
| 28 | The health App neither presents no sort of known susceptibility nor any type of malicious code. |
| 29 | The health App describes the security procedures established in order to avoid unauthorised access to personal data collected, as well as to limit the access by third parties. |
| 30 | The health App has encryption mechanisms for the storage and exchange of information, as well as mechanisms for passwords management. |
| 31 | When the health App uses services from the Cloud (cloud computing), the terms and conditions of those services are declared, and the pertinent security measures are ensured. |

**European Comission (European Union)**

| 1 | Be aware of, and comply with, their obligations as data controllers when they process data from and about users |
| --- | --- |
| 2 | Be aware of, and comply with, their obligations as data controllers when they contract with data processors such as if they outsource the collection and processing of personal data to developers, programmers and for example cloud storage providers |
| 3 | Ask for consent before the app starts to retrieve or place information on the device, i.e., before installation of the app. Such consent has to be freely given, specific and informed |
| 4 | Ask for granular consent for each type of data the app will access; at least for the categories Location, Contacts, Unique Device Identifier, Identity of the data subject, Identity of the phone, Credit card and payment data, Telephony and SMS, Browsing history, Email, Social networks credentials and Biometrics |
| 5 | Be aware that consent does not legitimise excessive or disproportionate data processing |
| 6 | Provide well-defined and comprehensible purposes of the data processing in advance to installation of the app, and not change these purposes without renewed consent; provide comprehensive information if the data will be used for third party purposes, such as advertising or analytics |
| 7 | Allow users to revoke their consent and uninstall the app, and delete data where appropriate |
| 8 | Respect the principle of data minimisation and only collect those data that are strictly necessary to perform the desired functionality |
| 9 | Take the necessary organisational and technical measures to ensure the protection of the personal data they process, at all stages of the design and implementation of the app (privacy by design) |
| 10 | Provide a single point of contact for the users of the app |
| 11 | Informs users about: who they are (identity and contact details) |
| 12 | Informs users about: what precise categories of personal data the app wants to collect and process |
| 13 | Informs users about: why the data processing is necessary (for what precise purposes) |
| 14 | Informs users about: whether data will be disclosed to third parties (not just a generic but a specific description to whom the data will be disclosed) |
| 15 | Informs users about: what rights users have, in terms of withdrawal of consent and deletion of data |
| 16 | Enable app users to exercise their rights of access, rectification, erasure and their right to object to data processing and inform them about the existence of these mechanisms |
| 17 | Define a reasonable retention period for data collected with the app and predefine a period of inactivity after which the account will be treated as expired |
| 18 | With regard to apps aimed at children: pay attention to the age limit defining children or minors in national legislation, choose the most restrictive data processing approach in full respect of the principles of data minimization and purpose limitation, refrain from processing children's data for behavioural advertising purposes, either directly or indirectly and refrain from collecting data through the children about their relatives and/or friends |
| 19 | Study the relevant guidelines with regard to specific security risks and measures |
| 20 | Proactively inform users about personal data breaches along the lines of the requirements of the ePrivacy Directive |
| 21 | Inform users about their proportionality considerations for the types of data collected or accessed on the device, the retention periods of the data and the applied security measures |
| 22 | Develop tools to enable users to customise retention periods for their personal data based on their specific preferences and contexts, rather than offering pre-defined retention terms |
| 23 | Include information in their privacy policy dedicated to European users |
| 24 | Develop and implement simple but secure online access tools for users, without collecting additional excessive personal data |
| 25 | Together with the OS and device manufacturers and app stores use their creative talent to develop innovative solutions to adequately inform users on mobile devices, for example through a system of layered information notices combined with meaningful icons |
| 26 | Be aware of, and comply with, their obligations as data controllers when they process data from and about users |
| 27 | Enforce the information obligation of the app developer, including the types of data the app is able to access and for what purposes, as well as whether the data is shared with third parties |
| 28 | Give special attention to apps directed at children to protect against the unlawful processing of their data, and especially enforce the obligation to present the relevant information in a simple manner, in age specific language |
| 29 | Provide detailed information on the app submission checks they actually perform, including those aimed to assess privacy and data protection issues |
| 30 | In collaboration with the OS manufacturer, develop control tools for users, such as symbols representing access to data on and generated by the mobile device |
| 31 | Subject all apps to a public reputation mechanism |
| 32 | Implement a privacy friendly remote uninstall mechanism |
| 33 | Provide feedback channels to users to report privacy and/or security problems |
| 34 | Collaborate with app developers to pro-actively inform users about personal data breaches |
| 35 | Warn app developers about the specificities of European law before submitting the application in Europe, for example about the consent requirement and in case of transfers of personal data to non-EU countries |
| 36 | Update their APIs, store rules and user interfaces to offer users sufficient control to exercise valid consent over the data processed by apps |
| 37 | Implement consent collection mechanisms in their OS at the first launch of the app or the first time the app attempts to access one of the categories of data that have significant impact on privacy |
| 38 | Employ privacy by design principles to prevent secret monitoring of the user |
| 39 | Ensure security of processing |
| 40 | Ensure (the default settings of) pre-installed apps are compliant with European data protection law |
| 41 | Offer granular access to data, sensors and services, in order to ensure that the app developer can only access those data that are necessary for his app |
| 42 | Provide user-friendly and effective means to avoid being tracked by advertisers and any other third party. The default settings must be such as to avoid any tracking |
| 43 | Ensure the availability of appropriate mechanisms to inform and educate the end user about what the apps can do and what data they are able to access |
| 44 | Ensure that each access to a category of data is reflected in the information of the user before the app’s installation : the categories presented must be clear and comprehensible |
| 45 | Implement a security-friendly environment, with tools to prevent malicious apps from spreading and allow each functionality to be installed/uninstalled easily |
| 46 | Enable users to uninstall apps, and provide a signal (for example through the API) to the app developer to enable deletion of the relevant user data |
| 47 | Systematically offer and facilitate regular security updates |
| 48 | Ensure that methods and functions allowing access to personal data include features aiming to implement granular consent requests |
| 49 | Actively help develop and facilitate icons alerting users to different data usage by apps |
| 50 | Develop clear audit trails into the devices such that end users can clearly see which apps have been accessing data on their devices and the amounts of outgoing traffic per app, in relation to user-initiated traffic |
| 51 | Be aware of, and comply with, their obligations as data controllers when they process personal data about users |
| 52 | Comply with the consent requirement determined in Article 5(3) of the ePrivacy Directive when they read or write data on mobile devices, in cooperation with the app developers and/or app stores, which essentially provide user with the information on the purposes of data processing |
| 53 | Not circumvent any mechanism designed to avoid tracking, as it currently often happens with the "Do Not Track" mechanisms implemented in browsers |
| 54 | Communications service providers, when they issue branded devices, must ensure the valid consent of users for pre installed apps and take on board relevant responsibilities when contributing to determining certain features of the device and of the OS, e.g. when limiting the user's access to certain configuration parameters or filtering fix releases (security and functional ones) provided by the device and OS manufacturers |
| 55 | Advertising parties must specifically avoid delivering ads outside the context of the app. Examples are delivering ads by modifying browser settings or placing icons on the mobile desktop. Refrain from the use of unique device or subscriber identifiers for the purpose of tracking |
| 56 | Refrain from processing children's data for behavioural advertising purposes, either directly or indirectly. Apply appropriate security measures. This includes secure transmission and encrypted storage of unique device and app user identifiers and other personal data |
| 57 | Develop and implement simple but secure online access tools for users, without collecting additional excessive personal data |
| 58 | Only collect and process data that are consistent with the context where the user provides the data |

**National Health Service (United Kingdom)**

| 1 | Is the health and/or wellbeing purpose clearly defined within the digital service? |
| --- | --- |
| 2 | Is it clearly stated within the service who it is suitable for and, if relevant, who it is not suitable for? |
| 3 | Is it clearly stated in the service when and how the digital service should be used in order to gain the health and care benefits claimed? |
| 4 | What type of evidence informed the design and development of the service? |
| 5 | Provide links to the evidence and describe how you identified it and how it was used. |
| 6 | Please list any ongoing studies that have not been previously disclosed as part of the app assessment process and which are designed to assess the benefit(s) of the digital service. |
| 7 | Does your digital service make anonymous data available to other systems? |
| 8 | How do you ensure that no identifiable data is provided? |
| 9 | Does your digital service make pseudonymised data available to other systems? |
| 10 | How do you ensure data is adequately pseudonymised? |
| 11 | If the digital service collects or processes personal data (e.g. user's name and address; personal email address) does this also include sensitive data e.g. physical or mental health or condition; the racial or ethnic origin of the user? |
| 12 | What is the legal basis for the processing of Personal data which is described, e.g. in a privacy notice, to the user? |
| 13 | What is the legal basis for your processing of SENSITIVE Personal data which is described, e.g. in a privacy notice, to the user? |
| 14 | Please evidence how valid consent is obtained from young people under the age of 16 or those without capacity to give consent Please describe how valid explicit consent, requiring a positive action by the user, is obtained and recorded. |
| 15 | Is the collection and use of personal data and sensitive personal data made fair and transparent to the user? |
| 16 | Are you able to respond to the users' legal rights to access their personal information i.e. a written request (from the user or a third party entitled to act on behalf of the individual) e.g. provide the users with copies of their personal data you hold on them when requested? |
| 17 | Is the personal data and/or sensitive personal data collected the minimum required for the purposes described to the user e.g. in a privacy notice, and for which a legal basis e.g. consent, for the processing exists |
| 18 | When the privacy policy is changed, are the changes brought to the attention of the user? |
| 19 | Does your service enable the user to delete, or request that you delete, all their personal data on request, irrespective of where the data is held? |
| 20 | Do you have a data retention policy? |
| 21 | Does this policy set out retention periods that ensure personal data processed for the purpose(s) described to the user, is not be kept for longer than is necessary for that purpose(s)? |
| 22 | Are encrypted connections used for transmission of personal data? What encryption standards do you use? |
| 23 | Do you use encryption to store personally identifiable data? What encryption standards do you use? |
| 24 | Is sent data appropriately safeguarded? Please state what the safeguards are |
| 25 | Are any and all third party components used such as libraries and frameworks identified and checked for known vulnerabilities? |
| 26 | Are security controls are enforced only on the client side or are they also enforced on the respective remote endpoints as well. |
| 27 | What system credential storage facilities are used to store sensitive data, such as user credentials or cryptographic keys? |
| 28 | Is sensitive data written to application logs? |
| 29 | Is the keyboard cache disabled on text inputs that process sensitive data? |
| 30 | Is the clipboard deactivated on text fields that may contain sensitive data? |
| 31 | Could sensitive data be exposed via any interprocess communication mechanisms? |
| 32 | Could sensitive data, such as passwords and credit card numbers, be exposed through the user interface or leaked to screenshots? |
| 33 | Does the app/web service re-use the same cryptographic key for multiple purposes? |
| 34 | How does the remote endpoint authenticate client requests? |
| 35 | Does a password policy for the app/web service exist? (If yes) Is the password policy enforced at the remote endpoint? |
| 36 | How does the app manage incorrect authentication credentials been submitted an excessive number of times? |
| 37 | Is the app/web service code signed and provisioned with a valid certificate? |
| 38 | Describe the exception handling processes within the app/web service |
| 39 | Has a threat model for the app/web service and the associated remote services been produced that identifies potential threats and countermeasures. |
| 40 | Have all third party components have been assessed (associated risks) before being used or implemented? |
| 41 | Outline what process is in place to ensure that each time a security update for a third party component is published, the change is inspected and the risk evaluated? |
| 42 | Do remote endpoints verify that connecting clients use an up-to-date version of the mobile app? |
| 43 | Security is addressed within all parts of the software development lifecycle. |
| 44 | When placed in the background, does the app remove sensitive data from views? |
| 45 | Is sensitive data held in memory for only as long as is necessary? |
| 46 | Does the app/web service indicate to the user the security best practices the user should follow when using the app/web service? |
| 47 | What happens to sessions after a period of inactivity? |
| 48 | Does the app/web service inform the user of all login activities with his or her account? |
| 49 | Does the app/web service allow users to manage the list of devices used to access their account? (if yes) Can users block specific devices from accessing their account? |
| 50 | What communication channels (email or SMS) are used for critical operations, such as enrolments and account recovery? |
| 51 | Does the app detect whether it is being executed on a rooted or jailbroken device? (If yes) what actions are performed on execution of the app on a rooted or jailbroken device? |
| 52 | Does the app provide a custom keyboard whenever sensitive data is entered? |
| 53 | Are custom UI components used to display sensitive data? |
| 54 | Does the app/web service implement multiple functionally independent debugging defences that, in context of the overall protection scheme, force adversaries to invest considerable manual effort to enable debugging? |
| 55 | Does the app/web service detect, and respond to, tampering with executable files and critical data? |
| 56 | Does the app/web service detect the presence of widely used reverse engineering tools, such as code injection tools, hooking frameworks and debugging servers? |
| 57 | Does the app/web service detect modifications of process memory, such as relocation table patches and injected code? (if yes) How does the app/web service respond to modifications of process memory? |
| 58 | Are all executable files and libraries belonging to the app/web service either encrypted on the file level and/or important code and data segments inside the executables encrypted or packed? |
| 59 | Are any obfuscating transformations and functional defences interdependent and well-integrated throughout the app/web service? |
| 60 | Does the app implement a 'device binding' functionality using a device fingerprint derived from multiple properties unique to the device? |
| 61 | Does the architecture require sensitive information be stored on the device? (if yes) Does the app only run on operating system versions and devices that offer hardware-backed key storage? Alternatively, Is the information protected using obfuscation? |
| 62 | Did the user centred design (UCD) process follow a set of well defined planned phases - for example discovery, alpha, beta, go live? |
| 63 | Was a clear user demographic defined at the outset of app development? |
| 64 | Was a representative/suitable sample of this user demographic engaged with throughout the user centred design process? |
| 65 | For each of the user needs were clear user acceptance criteria defined? |
| 66 | Were early versions of the app evaluated with a sample of the user demographic? |
| 67 | Throughout the evaluation of early versions and pre-release versions were changes made to the app in light of the user feedback? |
| 68 | Post-release do you continue to collect feedback from users and make changes to the app based on this feedback? |
| 69 | Is there an continuous process planned of improving the app through user centred design? |
| 70 | Do you use recognised standards based data structures and formats to store and represent data within your system? Which data structures or standards do you use? |
| 71 | Do you allow 3rd party access to data you store (whether collected or subsequently generated by you, or not)? |
| 72 | Do you support multiple versions of your data structures or formats, for compatibility or other reasons? |
| 73 | Do you have defined processes for responding to changes to data structures, formats or access arrangements? |
| 74 | Do you use services published by 3rd parties? |
| 75 | Do you have steps to protect your service, data or your users’ experience against such events? Please explain further |
| 76 | The source code and any configuration items for the digital service MUST be kept under version control with all changes audited. |
| 77 | There MUST be an active system to accept and respond to reports of technical faults from end users? |
| 78 | There MUST be capability to rollback to previous versions of the digital service in the case of significant problems following an update. (if Yes) Please provide documentation to support this |

**TIC Salut Social Foundation (Spain)**

| 1 | The main elements (text, images, icons, buttons, etc.) are identifiable and easy to use |
| --- | --- |
| 2 | The registry form is quickly for filling (it does not contain more than five fields) |
| 3 | The text font is understandable and easy to read (size, color, and font) |
| 4 | There are options to sound alerts: visuals or by vibrating |
| 5 | The fields registry format are simple and open (without number limitation of characters, capital letters, etc.) |
| 6 | The data entry is highlighted if possible |
| 7 | Graphics resources are used as color inversion for highlighting a chosen item |
| 8 | It admits and display all proper international characters correctly |
| 9 | The access process to the service is performed nimbly and quickly (by sending a confirmation mail and access data validation, etc.) |
| 10 | The functionality is adapted to the purpose/target of the application |
| 11 | The navigation is intuitive |
| 12 | The steps to follow are simple and make sense |
| 13 | Tabs use, menus, and options easy the navigation and bring inputs about where is the user |
| 14 | Not correctly filled up or the incorrect fields are clearly displayed |
| 15 | The registry areas add support mechanisms to easy the process (default schedules drop-downs fields, descriptive, etc.) |
| 16 | The operational entry data are clearly indicated |
| 17 | The features (GPS, sensors, etc.) work correctly |
| 18 | The features integrated into the App are quick to load in reasonable ranges of time |
| 19 | The position, size, ranges and element distances are correct to use it intuitively readable and efficient |
| 20 | Each element function is clearly identified (clickable, static, drop down, selector, video, etc.) |
| 21 | The App visual icons are understandable and visibly show the related features |
| 22 | It adds a backward button for returning directly to the home page and the previous page |
| 23 | It minimizes the steps to perform by the user accessing to any option (recommended: maximum 3) |
| 24 | It handles the multiple taps or multi touch correctly |
| 25 | In any case, the App is readable, and the text length is reasonable |
| 26 | If there is more than one language, language shift works, and it can adjust itself to the appropriate shape to the contents interface |
| 27 | It can correctly handle all the devices issues, precision error, hardware errors from an inaccurate usage |
| 28 | The user is notified to confirm orders which have devastating effects |
| 29 | Users are warned about a potentially serious error |
| 30 | It notifies the user if it is required a long boot time (up to 5 seconds) |
| 31 | It alerts the user if there are long lasting procedures |
| 32 | It allows the user to cancel long-lasting procedures |
| 33 | It warns in case of non-connectivity when the user requires the use of the App |
| 34 | It notifies the user of low-quality network to which it is being connected if necessary for the App usage |
| 35 | The screen effect works correctly for the device position change, emerging menus, emerging windows, etc. |
| 36 | The appropriate keyboard for each type of entry is displayed |
| 37 | The information architecture of the application is symmetric, harmonic and proportioned |
| 38 | If the user accepts an incoming call while the app is working, it should be able to return to the same point after the call terminates |
| 39 | Often the system helps to use figures or graphics |
| 40 | Colour Coding is uniform and aesthetically coherent following a pattern |
| 41 | All graphic elements (typographies, icons, buttons, etc.) are used in the same way in all views. There is a consistency |
| 42 | It includes visual icons adding a nice looking to the application |
| 43 | It combines equally audio-visual contents with text contents |
| 44 | The App is well placed (texts for each different language, currency, spelling mistakes,...) |
| 45 | The elements color contrast with background should be enough (minimum 4,5:1) |
| 46 | The application is consistent with products support (screen reader, magnifiers, voice commands, etc.) |
| 47 | The app matches with accessibility tools of the operating system (VoiceOver, Zoom, Invert Colors, Bold text, etc.) |
| 48 | Controls, objects, icons, and images must have an alternative related text indicating its function or meaning (labels) |
| 49 | It verifies the mistaken data entry (format, range, etc.) |
| 50 | It does not fail immediately during its use (blocks, etc.) |
| 51 | It is retrieved correctly in context changes (switch to other app and return), external interruptions (incoming call or message, etc.) and switched off the terminal |
| 52 | It does not waste resources excessively: battery, CPU, memory, data, etc. |
| 53 | Installed and uninstalled correctly |
| 54 | It does not fail if it is used during long-time |
| 55 | When the App is running in background, this does not affect the function system or other applications, unless it is designed for this in particular |
| 56 | The database resources must be appropriately shared with the app and the system |
| 57 | It does not need to use excessively network resources unnecessarily |
| 58 | The application speed is acceptable for the required purpose, and it must not alter the user experience or be uncontrollable |
| 59 | It behaves correctly running within one-hour event during its performance |
| 60 | App’s repeated suspension and return are correctly controlled |
| 61 | By using its network capabilities, it can work in flight mode |
| 62 | By using the network capacities, it can deal with network delays and any loss of connection |
| 63 | It can work in pause mode and resume the downloads interruptions during the use of network capabilities for downloading resource files |
| 64 | It is resumed from the suspension status when a scheduled event is expired |
| 65 | A scheduled event is correctly initiated after escaping from the App. |
| 66 | Never blocked or freezing while the device is being executed |
| 67 | It reports on the need of the user App data registry for its performance |
| 68 | It asks the necessary permissions to access to different device services, and these are disclosed and described |
| 69 | The app has a password recovery mechanism |
| 70 | The app produces errors log or actions monitoring in an external system |
| 71 | It reports about the user data typology gathered during its performance |
| 72 | It informs about the purpose and use that the owner will do with registered data |
| 73 | It does not collect data unless they are essential for the service delivery perceived by the user |
| 74 | It specifies if third bodies will access to data, as well as the conditions of this transfer will be done |
| 75 | It describes the maintenance policy and the data erasure provided by the user |
| 76 | It performs the data user authentication (for instance, professional associations) correctly |
| 77 | It describes access privileges, rectification, void or data cancellation |
| 78 | The passwords are note directly displayed and are not gathered in the device without encrypting |
| 79 | The personal information access is managed correctly with the user approval |
| 80 | It has an own user validation mechanism to access to the confidential or private information |
| 81 | The communication channels used are encrypted if are sensitive data |
| 82 | The authorisation and authentication mechanisms are done correctly |
| 83 | The binary fields of the app are blurred |
| 84 | The authentication mechanism is a Standard as Basic, OAuth, OpenID, etc (new) |
| 85 | The system, if it allows recovering the password, creates a new one because the passwords are gathered encrypted in a database. |
| 86 | The system allows to modify/regenerate the password in the event of loss or user’s decision |
| 87 | The App will not record the device credentials |
| 88 | The App will not record the encrypted device credentials |
| 89 | If the App creates error logs on the device, they are encrypted |
| 90 | The server uses a valid encrypt certification and created by a certified body (no self- signed) |
| 91 | They are explicitly indicated or actually intuitable (the name identifies the app, etc.) the audience to whom this APP Is set out |
| 92 | APP usage benefits or advantages are indicated |
| 93 | The contents offered are attractive to the user profile to whom it is set out |
| 94 | The functions enlisted are useful for users, regarding saving time or having further information |
| 95 | A plain and understandable language is used, with messages adapted to the user's profile regarding linguistic level or style |
| 96 | It is clearly identifiable whose is the App |
| 97 | Additional information about the App’s owner is displayed (by an external link or inside the APP itself) |
| 98 | Third bodies and/or organizations contributors for the App development are clearly identified |
| 99 | App financial, promotion and sponsorship sources are reported |
| 100 | It is clearly identified who is or are the App content responsible |
| 101 | A group of identified specialized professionals or a health department or a scientific society have joined the App content development |
| 102 | Additional information about the content authors is brought to foster credibility and to offer quality guarantees |
| 103 | Data used are suitable for the final user requirements |
| 104 | The data system used are liable and of renowned validity |
| 105 | The methodology to be used for generating contents is told |
| 106 | Information sources of the App gathered contents are shown |
| 107 | The Health advice provided by the App on gathered data basis are fitted to scientific proof |
| 108 | It is shown the frequency to revise or update the contents to the App |
| 109 | The last App revision date is disclosed in the App or the official store |
| 110 | It is warned that the App does not intend to replace the services provided by a professional |
| 111 | Potential risks arisen from a bad App usage and/or possible adverse effects are advised |
| 112 | The App advises that it be managing information/minors’ data |
| 113 | The App advises that it be managing information/third parties’ data |
| 114 | There are self-help elements as video tutorials, guides or FAQs to ease its usage |
| 115 | There are users’ support mechanisms (e-mail, phone, contact form) to solve doubts, problems or issues related to the health contents and technical support |

**Regulations**

**Mobile Medical Applications: Guidance for Food and Drug (United States)**

| 1 | Limit access to devices through the authentication of users (e.g. user ID and password, smartcard, biometric) |
| --- | --- |
| 2 | Use automatic timed methods to terminate sessions within the system where appropriate for the use environment |
| 3 | Where appropriate, employ a layered authorization model by differentiating privileges based on the user role (e.g. caregiver, system administrator) or device role |
| 4 | Use appropriate authentication (e.g. multi-factor authentication to permit privileged device access to system administrators, service technicians, maintenance personnel) |
| 5 | Strengthen password protection by avoiding “hardcoded” password or common words (i.e. passwords which are the same for each device, difficult to change, and vulnerable to public disclosure) and limit public access to passwords used for privileged device access |
| 6 | Where appropriate, provide physical locks on devices and their communication ports to minimize tampering |
| 7 | Require user authentication or other appropriate controls before permitting software or firmware updates, including those affecting the operating system, applications, and anti-malware |
| 8 | Restrict software or firmware updates to authenticated code. One authentication method manufacturers may consider is code signature verification |
| 9 | Use systematic procedures for authorized users to download version identifiable software and firmware from the manufacturer |
| 10 | Ensure capability of secure data transfer to and from the device, and when appropriate, use methods for encryption |
| 11 | Implement features that allow for security compromises to be detected, recognized, logged, timed, and acted upon during normal use |
| 12 | Develop and provide information to the end user concerning appropriate actions to take upon detection of a cybersecurity event |
| 13 | Implement device features that protect critical functionality, even when the device’s cybersecurity has been compromised |
| 14 | Provide methods for retention and recovery of device configuration by an authenticated privileged user |
| 15 | Monitoring cybersecurity information sources for identification and detection of cybersecurity vulnerabilities and risk |
| 16 | Maintaining robust software lifecycle processes that include mechanisms for monitoring third party software components for new vulnerabilities throughout the device’s total product lifecycle |
| 17 | Maintaining robust software lifecycle processes that include mechanisms for design verification and validation for software updates and patches that are used to remediate vulnerabilities, including those related to Off-the-shelf software |
| 18 | Understanding, assessing and detecting presence and impact of a vulnerability |
| 19 | Establishing and communicating processes for vulnerability intake and handling |
| 20 | Using threat modeling to clearly define how to maintain safety and essential performance of a device by developing mitigations that protect, respond and recover from the cybersecurity risk |
| 21 | Adopting a coordinated vulnerability disclosure policy and practice. Vulnerability Disclosure which may be a useful resource for manufacturers |
| 22 | Deploying mitigations that address cybersecurity risk early and prior to exploitation |
| 23 | The device manufacturer who uses OTS software in your medical device) bear theresponsibility for the continued safe and effective performance of the medical device, including the performance of OTS software that is part of the device |
| 24 | You should validate all software design changes, including computer software changes to address cybersecurity vulnerabilities, according to an established protocol before approval and issuance. For most software changes intended to address cybersecurity vulnerabilities, analysis, inspection, and testing should be adequate and clinical validation should not be necessary. |
| 25 | Maintain formal business relationships with your OTS software vendors to ensure timely receipt of information concerning quality problems and recommended corrective and preventive actions |
| 26 | Develop a single cybersecurity maintenance plan to address compliance with the regulation |
| 27 | Your software maintenance plan should provide a mechanism for you to exercise overall responsibility while delegating specific tasks to other parties |
| 28 | If the software patch affects the safety or effectiveness of the medical device, you should report the correction to FDA, even if a software maintenance plan is in effect |
| 29 | Manufacturers of medical devices are required to annually register their establishmentswith FDA and provide a list of the devices they market |
| 30 | Clinical studies with devices of significant risk must be approved by FDA and by an Institutional Review Board (IRB) before the study can begin. Studies with devices of non-significant risk must be approved by the IRB only before the study can begin. |
| 31 | Medical device manufacturers are required to comply with applicable labeling regulations for in vitro diagnostic products. |
| 32 | Mobile medical app manufacturers should identify the current classification covering their mobile medical app. |
| 33 | Mobile medical app manufacturers are required to develop requirements for their products that will result in devices that are safe and effective, and to establish methods and procedures to design, produce, and distribute their devices. |
| 34 | Appropriately verify and validate their mobile medical apps along with the mobile platform to ensure safe and effective operation of the mobile medical app. |
| 35 | Mobile medical app manufacturers are required to ensure that adequate controls and processes are in place through purchasing controls to ensure safe distribution, installation, and operation of the mobile medical app. |
| 36 | The Medical Device Reporting (MDR) regulation requires manufacturers and importers of medical devices to submit reports to the FDA whenever they receive or otherwise become aware of information, from any source, that reasonably suggests that a device they market may have caused or contributed to a death or serious injury, or has malfunctioned and the device or a similar device that they market would be likely to cause or contribute to a reportable death or serious injury if the malfunction were to recur. |
| 37 | A mobile medical app manufacturer may voluntarily take action at any time or may be requested to take action by the FDA to correct problems. |
| 38 | Inspecting the device for problems |
| 39 | Repairing the device |
| 40 | Adjusting settings on the device |
| 41 | Upgrading software to reduce risk from a “bug” or unintended response |
| 42 | Mobile medical app manufacturers are required to report to FDA any corrections made to a mobile medical app to reduce a risk to health posed by the mobile medical app or to remedy a violation of the FD&C Act caused by the mobile medical app which may present a risk to health. |

**Regulation of medical software and mobile medical 'apps' (Australia)**

| 1 | The device will not compromise the clinical condition or safety of a patient, or the safety and health of the user of any other person, when the device is used on a patient under the conditions and for the purposes for which the device was intended and, if applicable, by a user with appropriate technical knowledge, experience, education or training |
| --- | --- |
| 2 | Any risks associated with the use of the device are: acceptable risks when weighed against the intended benefit to the patient; and compatible with a high level of protection of health and safety |
| 3 | The solutions adopted by the manufacturer for the design and construction of a medical device must conform with safety principles, having regard to the generally acknowledged state of the art. |
| 4 | Without limiting subclause (1), in selecting appropriate solutions for the design and construction of a medical device so as to minimise any risks associated with the use of the device, the manufacturer must: |
| 5 | A medical device must: Perform in the way intended by the manufacturer |
| 6 | Be designed, produced and packaged in a way that ensures that it is suitable for one or more of the purposes mentioned in the definition of medical device in subsection 41BD(1) of the Act. |
| 7 | The device is used within the period, indicated by the manufacturer, in which the device can be safely used |
| 8 | The device is not subjected to stresses that are outside the stresses that can occur during normal conditions of use |
| 9 | The device is regularly maintained and calibrated in accordance with the manufacturer’s instructions |
| 10 | A medical device must be designed, produced and packed in a way that ensures that the characteristics and performance of the device when it is being used for its intended purpose will not be adversely affected during transport and storage that is carried out taking account of the instructions and information provided by the manufacturer |
| 11 | The benefits to be gained from the use of a medical device for the performance intended by the manufacturer must outweigh any undesirable effects arising from its use. |
